# Chondroprotective effect of high-molecular-weight hyaluronic acid on osteoarthritic chondrocytes in a co-cultivation inflammation model with M1 macrophages

**DOI:** 10.1186/s12950-016-0139-y

**Published:** 2016-09-13

**Authors:** Christoph Bauer, Eugenia Niculescu-Morzsa, Vivek Jeyakumar, Daniela Kern, Stephan S. Späth, Stefan Nehrer

**Affiliations:** Center for Regenerative Medicine and Orthopedics, Department for Health Sciences and Biomedicine, Danube-University Krems, Dr.-Karl-Dorrek-Strasse 30, Krems, Austria

**Keywords:** Inflammation, Hyaluronic acid, Chondrocytes, Macrophages

## Abstract

**Background:**

Osteoarthritis (OA) is described by an imbalance between anabolic and catabolic processes in the affected joint. This dysregulation of metabolism affects not only chondrocytes within cartilage tissue but also the cells of the synovial membrane across the border of the joint. An important factor in OA is the low viscosity of the synovial fluid. High-molecular-weight hyaluronic acid (HA) can be used to increase the viscosity and also reduce inflammatory processes. The purpose was to establish an in vitro inflammation model and to evaluate the effects of high-molecular-weight HA in a co-cultivation inflammation model of osteoarthritic chondrocytes and M1 macrophages.

**Methods:**

For the establishment of the inflammation model THP-1 cells were, at first, differentiated to M0 macrophages and then activated to the M1 subtype after 5 days of resting period. Surface markers, cytokine release, and gene expression, were analyzed to examine the successful differentiation. In the inflammation model, the defined M1 macrophages were co-cultivated with osteoarthritic chondrocytes for 2 days, with and without the addition of 10 % HA and further analyzed for chondrogenic gene expression markers and the release of cytokines in the supernatant.

**Results:**

The differentiation and activation process was successful as M1 macrophages expressed higher levels of pro-inflammatory cytokines and specific genes. Similarly, the surface marker CD14 was significantly decreased compared to M0 macrophages. For the co-culture system, the analysis of gene expression showed that HA increased the expression of cartilage-specific genes while catabolic-encoding genes exhibited lower expression levels than the control group. This positive effect of HA was also demonstrated by the measurement of pro-inflammatory cytokines, as their level decreased.

**Conclusion:**

Our study implies that high-molecular-weight HA has a chondroprotective effect in the present co-cultivation inflammation model, as it decreases pro-inflammatory cytokines and increases anabolic factors.

## Background

The development of osteoarthritis (OA) primarily occurs in response to mechanical stress associated with obesity, trauma or genetic predisposition [1–3]. OA patients suffer from pain, disability and a dramatic reduction in quality of life. During degenerative processes in OA, a massive destruction of joint tissue, including bone, cartilage, and synovial membrane occurs and leads to aseptic inflammation that further prolongs the joint disease. Cartilage breakdown is then associated with the formation of osteophytes, subsequent degeneration of menisci and ligaments as well as inflammation of the synovium that is transformed into a fibrotic tissue with pannus formation [1, 2, 4]. Synovial inflammation or synovitis plays a significant role in the progression of OA and is accompanied by hyperplasia and infiltration of macrophages and lymphocytes [5, 6]. These cells, as well as chondrocytes, produce soluble inflammatory mediators like IL-1β, IL-6, and TNF-α, which stimulate the production of degradative enzymes (e.g. MMPs, aggrecanases), that are often found in increased levels in OA synovial fluids post-injury in joint tissues [7–9].

**Table 1:** Sequences of Primers and conditions used in quantitative reverse transcriptase-polymerase chain reaction (RT-qPCR)

Primer	Abbreviation	Sequence (3′ – 5′)
Glyceraldehyde-3-phophate Dehydrogenase	GAPDH	
Sense Antisense		ctctgctcctcctgttcgac acgaccaaatccgttgactc
Aggrecan core protein 1	ACAN	
Sense Antisense		cctccccttcacgtgtaaaa gctccgcttctgtagtctgc
Collagen, type II, alpha 1	COL2A1	
Sense Antisense		gtgtcagggccaggatgt tcccagtgtcacagacacagat
Collagen, type I, alpha 1	COL1A1	
Sense Antisense		gggattccctggacctaaag ggaacacctcgctctccag
Matrix metalloproteinase 3	MMP3	
Sense Antisense		caaaacatatttctttgtagaggacaa ttcagetattcgcttgggaaa
Matrix metalloproteinase 9	MMP9	
Sense Antisense		cgaactttgacagcgacaag gccacgaggaacaaactgtat
Matrix metalloproteinase 13	MMP13	
Sense Antisense		tttcctcctgggccaaat gcaacaagaaacaagttgtagcc
Inducible nitric oxide synthase 2	iNOS	
Sense Antisense		gaccagtacgtttggcaatg tttcagcatgaagagcgattt

Treatment modalities for OA include non-pharmacological (e.g. physiotherapy), pharmacological (e.g. hormones) or intra-articular (e.g. injection of hyaluronic acid) therapies [1, 2]. Disease-modifying anti-rheumatic drugs (DMARDs) or non-steroidal anti-inflammatory drugs (NSAIDs), which are used in OA are inadequate due to poor and patient-specific efficacy. Although DMARDs and NSAIDs decrease pain and inflammation, they often fail to inhibit cartilage degradation. Furthermore, efficacy is lost over time and so constantly increasing doses are required to ensure an effect, which often augments their toxicity and other side effects. Corticosteroids, currently available as alternative treatment options are highly effective to reduce inflammation and pain by inhibition of the cytokine signaling mediators, but their adverse effects like osteoporosis or immunosuppression are highly problematic [10].

The mentioned therapies have been proved to reverse the symptoms in most cases. However, the potential to stop degeneration process of the cartilage and in promoting the reconstruction of the tissue is limited. Therefore, the development of new therapies with disease-modulating aspects, like the reduction of pro-inflammatory cytokines and degrading enzymes play a central role in osteoarthritis research with a substantial increase in cost effectiveness [1, 2]. In this context, hyaluronic acid (HA), used as an intra-articular treatment option, is a conventional therapy to replace the synovial fluid that has lost its viscoelastic properties. In cases of inflammation and tissue injury, HA plays a central role, as it can function as a pro- and anti-inflammatory molecule. This fact highly depends on its molecular weight, microenvironment and the availability of specific binding partners [11–13].

Despite strong potentials of HA in tissue engineering, its precise role in anti-inflammatory responses on OA chondrocytes is not well explored.

In this study, we evaluated the influence of pro-inflammatory M1 macrophages on osteoarthritic chondrocytes in a newly established in vitro co-culture system and assess the chondroprotective effect of high-molecular-weight HA against an inflammatory response.

## Methods

### Hyaluronic acid

The high-molecular-weight HA is highly purified and has a molecular weight of 1600 kDA. It was produced by Croma Pharma GmbH by fermentation process and the raw material was further dissolved in PBS (phosphate buffered saline). The product is commercially available and widely used in clinical practice.

### Cell culture and differentiation of THP-1 monocytes to macrophages

The THP-1 monocytic cell line was obtained from ATCC. The cells were cultured at a density of 0.2 – 1.0 × 10^6^ cells/ml in culture medium (GIBCO® DMEM/F12 GlutaMAX ™-I, Invitrogen, LifeTech Austria, Vienna, Austria) with antibiotics (penicillin 200 U/ml; streptomycin 0.2 mg/ml and Amphotericin B 2.5 μg/ml (Sigma-Aldrich Chemie GmbH, Steinheim, Germany)). For the establishment of the inflammation model, THP-1 cells were seeded into 175 cm^2^ cell culture flasks (2.3 x 10^6^ cells/flask) and differentiated to macrophages for 3 days by adding 100 nM phorbol-12-myristate 13-acetate (PMA) (Sigma-Aldrich Chemie GmbH, Steinheim, Germany). After the initial stimulation, the differentiation of PMA treated THP-1 cells was extended by removing the PMA containing media and incubating the cells in fresh culture medium for further 5 days.

### Differentiation to M1 macrophages

After a total of 8 days (3 days PMA, 5 days resting), the obtained resting M0 macrophages (rM0) were activated to the M1 phenotype by adding 20 ng/ml IFN-γ (Sigma-Aldrich Chemie GmbH, Steinheim, Germany) and 500 ng/ml LPS (Sigma-Aldrich Chemie GmbH, Steinheim, Germany) to the fresh culture medium for 2 days.

### Co-cultivation of osteoarthritic chondrocytes and M1 macrophages

For the co-culture system, THP-1 cells were seeded onto cell culture inserts (0.6 × 10^5^ cells/Thincert™ cell culture insert, Greiner Bio-one, Kremsmuenster, Austria) for 6-well plates and differentiated to rM0 as described above. During differentiation process osteoarthritic chondrocytes were seeded onto 6-well plates before the co-cultivation was started by adding 10 % PBS or 10 % of a 1.0 mg/ml HA solution to the culture medium of the chondrocytes and activate the rM0 to the M1 subtype.

### Isolation and cultivation of human osteoarthritic chondrocytes

Human articular cartilage was received from the University Hospital Krems from 10 osteoarthritis patients undergoing total knee arthroplasty. Because of low cell numbers in most of the cartilage samples, only 3 patients (2 female, 1 male) which had an average of 67 years, approximately 75 to 85 kg and an average height of 180 cm could be used in the study. The patients were suffering osteoarthritis since 1 to 6 years and were treated by infiltration of the knee and Cortison or hyaluronic acid. In all cases informed consent was obtained, and the study was approved by the Regional Ethical Committee (GS4-EK-4/199-2013).

For chondrocyte isolation, articular cartilage was minced into 2 mm^3^ pieces prior to enzymatic digestion with Liberase TM (0.2 WU/ml, Roche Diagnostics GmbH, Mannheim, Germany) in medium (GIBCO® DMEM/F12 GlutaMAX ™-I, Invitrogen, LifeTech Austria, Vienna, Austria) with antibiotics (penicillin 200 U/ml; streptomycin 0.2 mg/ml and Amphotericin B 2.5 μg/ml (Sigma-Aldrich Chemie GmbH, Steinheim, Germany)) under permanent agitation for 18–22 h at 37 °C. The resulting chondrocyte suspension was passed through a Cell Strainer with 40 μm pores (BD, Franklin Lakes, NJ) to remove undigested debris, washed with phosphate-buffered saline (PBS), centrifuged (10 min, 500 g, room temperature [RT]) and resuspended in growth medium (i.e. medium supplemented with antibiotics (see above), 10 % FCS (PAA Laboratories GmbH, Linz, Austria) and 0.05 mg/ml ascorbic acid (Sigma-Aldrich Chemie GmbH, Steinheim, Germany)). Viability was determined via trypan blue (Sigma-Aldrich Chemie GmbH, Steinheim, Germany) staining and cells were counted using a hemocytometer.

The isolated cells were seeded in growth medium in 75 cm^2^ culture flasks (Nunc, Rochester, NY) at a density of 1×10^4^ cells/cm^2^ and cultivated at 37 °C in a humidified environment with 5 % CO_2_. The medium was changed every 2 to 3 days till 80 % confluency. After expansion, cells were harvested by the use of accutase (1.5 ml/flask; PAA Laboratories GmbH, Linz, Austria), counted and seeded in 6-well plates at a density of 1×10^4^ cells/cm^2^ (P1 cells).

### Flow cytometry

The expression levels of CD14 in the THP-1 cell line, resting M0 and M1 macrophages were assessed using flow cytometry (FC). For FC, 2 × 10^5^ cells were detached, fixed, incubated with IgG from human serum (Sigma-Aldrich Chemie GmbH, Steinheim, Germany) for 20 mins followed by incubation with anti-CD14 FITC mouse monoclonal antibody for 45 mins at room temperature in dark and washed twice with PBS. Flow cytometry analyzes were performed on samples using the Flow cytometer FC500 (Beckman Coulter, Brea, CA, USA).

### Quantification of proteins

The stored supernatants from THP-1 derived M1 macrophages and osteoarthritic Chondrocytes were analyzed for the level of cytokines. Cytokines including IL-1β, IL-6, and TNF-α were measured with Bio-Plex Pro Assays (BIO-RAD Laboratories, Inc., Hercules, CA, USA). In this human cytokine multiplex assay, antibodies are covalently coupled to magnetic beads with a unique fluorescent dye. Thereby the determination of concentrations of each analyte using the Bio-Plex 200 analyzer (BIO-RAD) is possible. For the purpose of analysis, values below the lower limit of detection for each analyte were recorded as the lower limit of quantification (LLOQ).

### Total-RNA isolation and reverse transcription

For gene expression analysis of THP-1 derived M1 macrophages and osteoarthritic Chondrocytes, the total-RNA from 6-Well-plates was extracted using the High Pure RNA Isolation Kit (Roche Diagnostics GmbH, Mannheim, Germany) in accordance with the manufacturer’s protocol. Complementary DNA (cDNA) was synthesized with 1st Strand cDNA Synthesis Kit for RT-PCR AMV (Roche Diagnostics GmbH, Mannheim, Germany) and Random Primer p(dN)6 according to the supplier’s instruction. cDNA was stored at −20 °C until it was used for real-time PCR.

### Quantitative reverse transcriptase-polymerase chain reaction "RT-qPCR"

Dual labeled probe-based RT-qPCR was performed with FastStart TaqMan® Probe Master (Roche Diagnostics GmbH, Mannheim, Germany) and with gene-specific primers (Eurofins MWG Synthesis GmbH, Ebersberg, Germany) in triplicate on the iCycler iQ (Bio-Rad Laboratories, Hercules, CA). Probes and primers were selected by use of Universal Probe Library System and by applying *in silico* PCR (Roche). The primer-dependent optimal annealing temperature was determined experimentally. RT-qPCR was carried out as follows: initial denaturation step at 95 °C for 10 minutes, further denaturation at 95 °C for 30 seconds, an annealing step at 55 °C to 62 °C optimized for the respective primers (Table 1) for 30 seconds, a polymerization step at 72 °C for 15 seconds. The data resulting from the fluorescence measurement were relatively quantified without efficiency correction with R = 2 ^– ∆Ct [MEAN target-MEAN reference]^ method [14].

### Statistical analysis

Each experiment was performed in triplicate. Data values are reported as the mean ± standard deviation. Statistical analysis was performed using ANOVA with a confidence level of 95 % or more.

## Results and discussion

### Establishment of the in vitro inflammation model

While complex in vitro inflammation models do exist for other inflammatory diseases (e.g. bowel disease or diabetic nephropathy) [15, 16], there is no simple and readily available osteoarthritis model. Hence, our first objective was to establish an easy, yet meaningful in vitro inflammatory osteoarthritis model, composed of differentiated pro-inflammatory macrophages and human osteoarthritic chondrocytes. Since primary tissue macrophages cannot be readily expanded ex vivo, THP-1 monocytic cell line was initially used to establish an inflammation model with a more resembled phenotype of human monocyte-derived macrophages (MDM), which were then differentiated to the M1 macrophage subtype. After successful differentiation, the inflammation model was used in a co-culture system to evaluate the influence of pro-inflammatory M1 macrophages on osteoarthritic chondrocytes and the addition of high-molecular-weight hyaluronic acid, as it can act as an anti-inflammatory mediator.

**Fig. 1:**
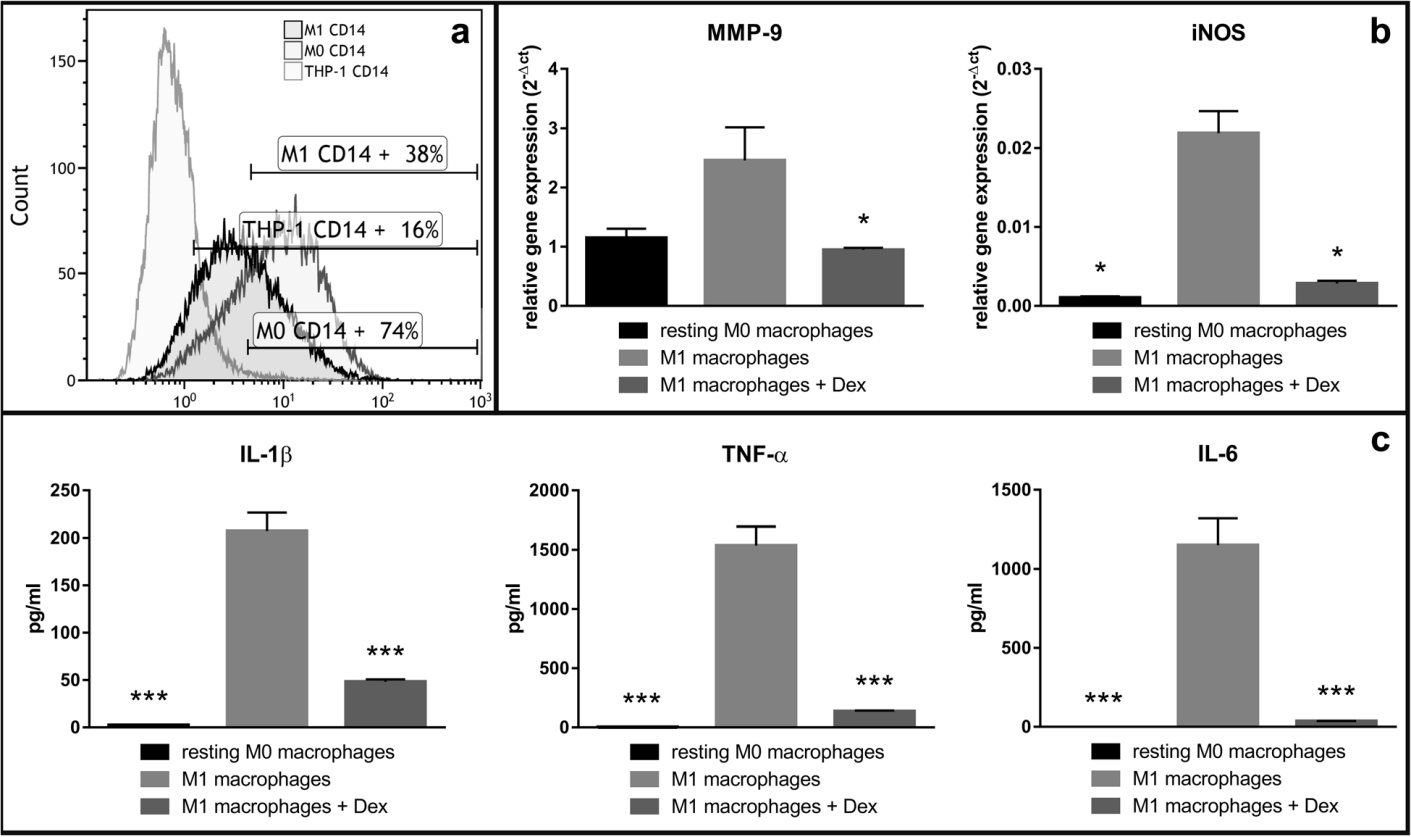
Establishment and verification of a functional in vitro macrophage inflammatory model. **a** Verification of the differentiation from THP-1 cells to resting M0 macrophages (rM0) and proinflammatory M1 macrophages via flow cytometry analysis of CD14 cell surface expression. **b** Representative RT-qPCR gene expression of M1-specific macrophage genes in rM0 and M1 macrophages (with or without the addition of the anti-inflammatory glucocorticoid dexamethasone (Dex)). **c** Analysis of secreted pro-inflammatory cytokines into the supernatant after rM0 and M1 culture (with or without the addition of the anti-inflammatory glucocorticoid dexamethasone (Dex))

The differentiation process of the THP-1 cell line to resting M0 macrophages (rM0) was based on Daigenault et al., 2010, since this results in a phenotype that is more similar to monocyte-derived-macrophages (MDM). The successful differentiation was demonstrated by the expression of the specific surface marker CD14. In THP-1 cells 16 % were CD14 positive, while after PMA treatment 74 % of the cells (resting M0 macrophages) had an expression of the surface marker CD14. For establishment of the inflammation model, differentiated rM0 were activated to the pro-inflammatory macrophage subtype M1. To confirm this activation, different parameters of rM0 and M1 were analyzed. Decrease in CD14 surface marker expression is a well-described phenomenon with pro-inflammatory M1 macrophages compared to rM0. However, flow cytometry analysis revealed that only 38 % of the M1 macrophages were CD14 positive (Fig. 1a).

Further evaluation showed a significantly higher release of IL-1β, TNF-α, and IL-6 pro-inflammatory cytokines in activated M1 macrophages, compared to rM0, where no or much less inflammatory cytokines were secreted into the culture medium (Fig. 1c). To verify and confirm this phenomenon, we also have added an anti-inflammatory glucocorticoid “dexamethasone” to the M1 macrophage culture, which should reverse or even stop the release of pro-inflammatory cytokines from M1 macrophages into the medium. Veritably, the addition of 1 μg/ml dexamethasone to the activation media resulted in a significant reduction of pro-inflammatory cytokine release of M1 macrophages, which was not significantly different to the values in the control group (rM0) (Fig. 1c).

Also, a notable upregulation of M1 macrophage-specific genes (i.e. MMP9 and iNOS) is detectable in inflammatory processes [17–19]. We hence evaluated the expression of these two genes in rM0 and M1 macrophages (with or without the addition of dexamethasone). Gene expression analysis revealed a significant increase in both genes examined, where MMP9 and iNOS were both significantly increased in M1, compared to rM0 and the anti-inflammatory group, where dexamethasone was added to the activation media. As expected, the relative gene expression in the anti-inflammatory group was reduced 2-fold for MMP9 or 10- to 20-fold for iNOS (Fig. 1c). The above results confirm the successful in vitro differentiation of THP-1 monocyte-derived cells into pro-inflammatory M1 macrophages.

#### Establishment of reliable in vitro M1 macrophage and human osteoarthritic chondrocyte co-culture system

To determine the optimal culture conditions some preliminary experiments were performed to find the most appropriate cell seeding density of M1 macrophages in the Transwell™ inserts. Also, the volume of media was optimized to balance the release of cytokines throughout the Transwell™ system to have an influence on the chondrocytes.

#### Presence of high-molecular-weight hyaluronic acid reduces pro-inflammatory cytokine secretion from human osteoarthritic chondrocytes

**Fig. 2:**
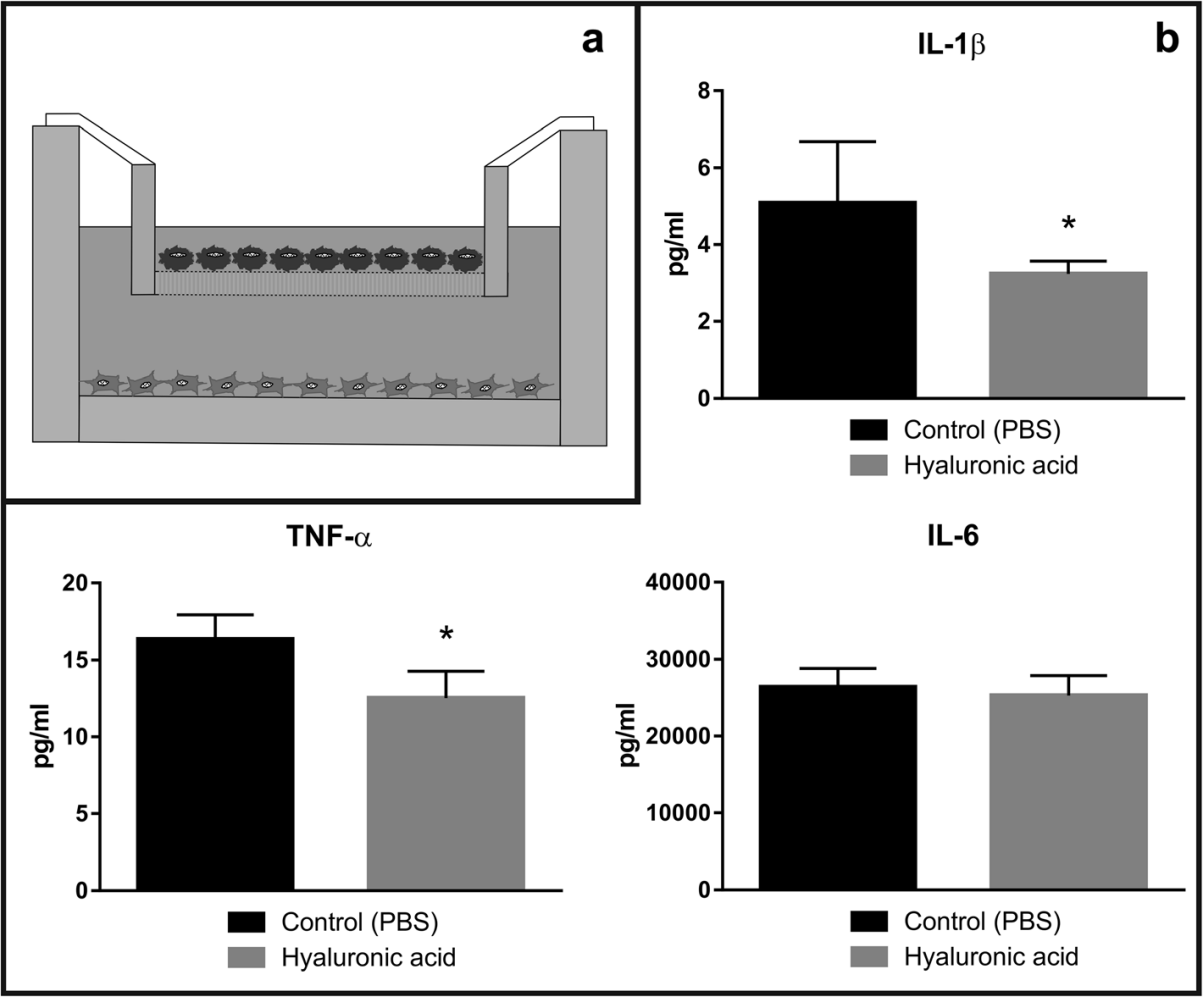
Effect of hyaluronic acid on pro-inflammatory cytokine secretion. **a** Schematic drawing of the in vitro inflammatory co-culture setup of differentiated monolayer M1 macrophages on the top and human monolayer osteoarthritic (OA) chondrocytes at the bottom. **b** Quantification of pro-inflammatory cytokine concentration in culture medium in the presence or absence of hyaluronic acid

The implication of hyaluronic acid (HA) as an anti-inflammatory agent in inflammatory diseases has been proposed and investigated in in vitro and in vivo conditions [20]. We intended to evaluate the role of high-molecular-weight (high-MW) HA on the inflammatory response on human osteoarthritic chondrocytes in our established in vitro co-culture system (Fig. 2a). Our results have shown that incubation of OA chondrocytes with high-MW HA (1600 kDa) leads to a reduced concentration of secreted IL-1ß and TNF-α cytokines into the medium, compared to the control group (Fig. 2b). We also observed a trend towards a lower concentration of secreted IL-6 for the HA-treated group, which however did not reach statistical significance. The above results possibly suggest the anti-inflammatory properties of high-MW HA or a potential activation of anti-inflammatory mechanisms in human OA chondrocytes.

#### High-molecular-weight hyaluronic acid increases anabolic and reduces catabolic processes in human osteoarthritic chondrocytes

**Fig. 3:**
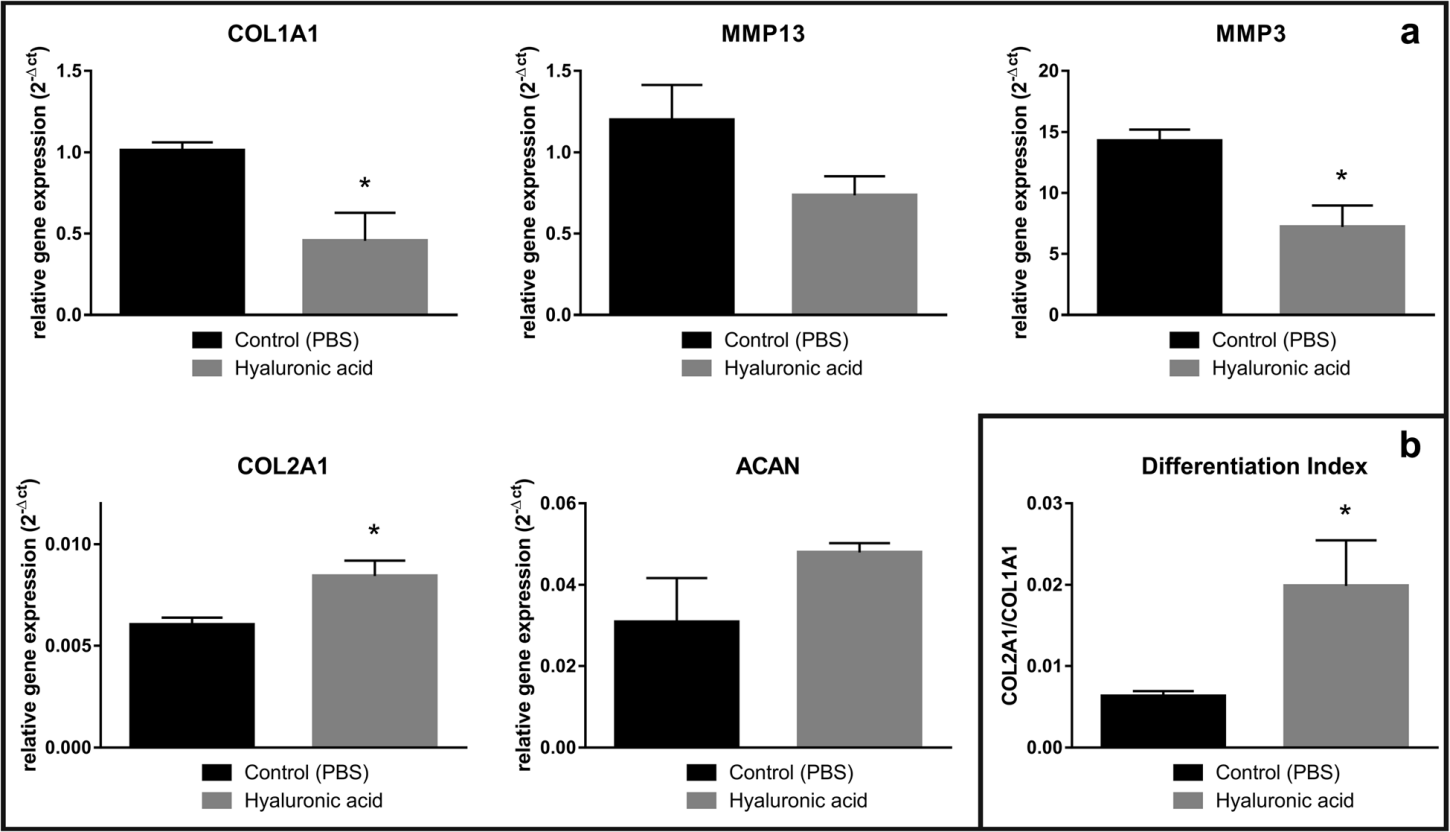
Effect of hyaluronic acid on gene expression in human osteoarthritic chondrocytes. **a** Representative RT-qPCR gene expression of anabolic and catabolic cartilage markers in human osteoarthritic monolayer chondrocytes in the co-culture system, with or without the addition of hyaluronic acid. **b** Comparative differentiation index between collagen type II and collagen type I in human osteoarthritic monolayer chondrocytes in the co-culture system, with or without the addition of hyaluronic acid

Anabolic and catabolic processes are significantly affected in osteoarthritis. In cartilage anabolic processes are marked by the presence of collagen II (COL2A1) and aggrecan (ACAN), which are major constituents of hyaline cartilage. Major markers of catabolism in OA cartilage are MMP13 and MMP3, which are known to be elevated. We hence intended to evaluate the effect of high-MW HA on these processes in human OA derived chondrocytes. Our results have revealed that the gene expression of COL2A1 was significantly increased by treating OA chondrocytes with high-MW HA in our established co-culture system (Fig. 3a). ACAN was also increased, however, did not reach statistical significance. On the other hand, catabolic mediators like matrix-degrading enzymes (i.e. MMP13 and MMP3) were decreased by adding the high-MW HA to the culture media, where the difference for MMP3 was even significant. Also, the positive effect of the used HA was also reflected by the gene expression of COL1A1, as it decreased by 3-fold compared to the control group. As a result, the differentiation index (ratio of COL2A1 to COL1A1) was significantly increased in the HA-treated group (Fig. 3b).

Despite the availability of several treatment options and surgical approaches to tackle osteoarthritis (OA), complications and side effects are still common among affected individuals [1, 2, 21–23]. Among several alterations in OA, such as inflammatory and biomechanical properties of cartilage, the reduction in lubricative properties of the synovial fluid, as well as its constituents (i.e. hyaluronic acid (HA)) are important factors [22, 24]. Preliminary observation and evaluation of conventional approaches, like intra-articular viscosupplemental injections of HA have shown some convincing benefit and anti-inflammatory potential over the past years in both animal and human subjects [13, 20, 22, 24–27]. In this study, we examined the anti-inflammatory, pro-anabolic and anti-catabolic properties of high-MW HA on human derived OA chondrocytes in an established and optimized in vitro pro-inflammatory macrophage co-culture model. In vitro inflammatory models are useful preliminary approaches to evaluate pharmacological or other biological agents before being tested on animals or in other in vivo conditions. Commonly existing in vitro inflammation models includes lung [28], intestine [29], brain [30], skin [31], but for OA, no simple, reproducible and easy handle in vitro inflammation model does exist. To study the biological effects of HA on human OA chondrocytes, we first established and optimized an in vitro inflammatory macrophage-human OA chondrocyte co-culture model. We chose the pro-inflammatory M1 macrophage and human OA chondrocytes in vitro transwell co-culture to be most appropriate, as it closely resembles the permeable synovial joint. The successful isolation of primary macrophages is difficult and not very reliable. Hence, we adapted our protocol based on Daigenault et al., 2010 in obtaining differentiated and activated pro-inflammatory M1 macrophages from human monocyte-derived THP-1 cell line [32]. We also characterized the successful differentiation process of the THP-1 cells to rM0 or pro-inflammatory M1 macrophages by flow cytometry and cytokine assay, utilizing the cell surface marker CD14 (Fig. 1a) and pro-inflammatory cytokines. We observed a much higher cell surface expression of CD14 in the resulting rM0 phenotype when THP-1 cells were treated with PMA, as it is described in literature [33]. Further differentiation with IFN-γ and LPS lead to a decrease in this surface marker, indicating a favorable differentiation towards M1 (Fig. 1a). These are well described phenomena. Several studies have shown that activated M1 macrophages secrete pro-inflammatory cytokines, like IL-1ß, TNF-α, and IL-6 [34, 35]. The presence of these cytokines is a commonly observed phenomenon in OA [24, 36, 37] and does closely mimic OA conditions in our established inflammation model. To further confirm the differentiation status of M1 macrophages, we treated differentiated M1 macrophages with the anti-inflammatory glucocorticoid agent dexamethasone, which suppresses the inflammatory response [38]. Indeed, we observed a significantly higher secretion of IL-1ß, TNF-α, and IL-6 from M1 macrophages that being further suppressed in the presence of dexamethasone, compared to resting M0 macrophages (Fig. 1c). It should also be noted that CD14 is a widely used marker for monocyte-macrophage differentiation [33]. But still some studies report a variation in the response of PMA to THP-1, as there is an increased CD14 level [39–41] or poor to no response [42]., The production of pro-inflammatory cytokines was used to confirm the presence of differentiated and activated M1 macrophages. The above data presents a robust and repeatable inflammatory model, consisting of differentiated pro-inflammatory M1 macrophages. To closely simulate the permeable OA synovial joint, we have optimized co-culture conditions of differentiated M1 macrophages, and human derived OA chondrocytes, utilizing the Transwell™ system. Hyaluronic acid (HA) is a major component of extracellular matrix (ECM), which is present in almost all tissues [20]. The different roles of HA are highly dependent on its molecular weight [43]. In the healthy joint, HA is an essential synovial fluid constituent with a high-molecular-weight (>1000 kDa) and moderate ionicity [44]. Besides anti-inflammatory characteristics (i.e. reduced prostaglandin release [45] and reduced oxidative stress [46]), it contributes to elasticity and viscosity of the synovial fluid, shock absorption and cartilage ECM structural and functional integrity [22]. HA exists as a polymer of various chain lengths and different crosslinking, but previous experiments indicate that HA molecular weights of 700–6000 kDa are best suited for cartilage repair [13, 44]. Despite the fact that low-MW (>10 kDa) HA has appropriate viscoelasticity, moisture retention, and adhesive properties, high-MW HA is used in clinical practice [13]. Many different HA preparations are commercially available in Europe and the USA, derived from different sources, with different molecular weights, joint residence time and rheological properties [47]. Due to the reasons explained above, we have evaluated the effect of high-MW HA on pro-inflammatory cytokine secretion and catabolic, as well as anabolic cartilage markers in cultured human OA chondrocytes, present in our established transwell co-culture (Fig. 2a). Our results have shown a significantly reduced secretion of pro-inflammatory IL-1ß and TNF-α cytokines, but not IL-6 in the HA-treated group, compared to the control group (Fig. 2b). Also, high-MW HA treatment of human OA chondrocytes in our co-culture system resulted in an increased gene expression of anabolic cartilage markers (i.e. COL2A1 and ACAN) and reduced catabolic cartilage markers (i.e. MMP13 and MMP3), compared to the control group (Fig. 3a). Chondrocytes tend to de-differentiate in prolonged 2D cultures, marked by fibroblastic morphology and the expression of collagen type I (COL1A1) [48, 49]. In our transwell co-culture, we observed a significant reduction of COL1A1 gene expression (Fig. 3a) and a significant increase in COL2A1/COL1A1 differentiation index in high-MW HA-treated human OA chondrocytes, compared to the non-treated control group (Fig. 3b). Our study confirms the beneficial effects of high-MW HA in previously reported in vitro and in vivo studies. Further, this study confirmed and strengthened previously reported anti-inflammatory, anti–catabolic and pro-anabolic properties of high-MW HA in OA chondrocytes and cartilage [13, 22, 24–27]. The role of HA and his fragments has been described for several biological processes (i.e. inflammation) and clinical conditions, but very limited information is available for HA in OA chondrocytes [50]. There is increasing evidence that HA fragmentation through inflammatory induction of hyaluronidases leads to worsening conditions in OA [44]. The inhibitory activity of high-MW HA on the secretion of pro-inflammatory cytokines (i.e. IL-1ß, TNF-α), MMP gene expression (i.e. MMP13 and MMP3) and no significant change in IL-6 expression, as observed in our study, is consistent with previously published results.

## Conclusions

This study evaluated the anti-inflammatory, catabolic and anabolic properties of high-MW HA in a robust in vitro transwell co-culture, containing differentiated pro-inflammatory M1 macrophages and human derived OA chondrocytes. Similar to previous studies, we observed a significant reduction in pro-inflammatory cytokine secretion and catabolic cartilage markers, as well as a statistical increase of anabolic cartilage markers and COL2A1/COL1A1 differentiation index. Finally, the results obtained from in vitro models, as in our setting, should be treated with extreme caution and vigorously confirmed with subsequent complex follow-up in vivo experiments, before being applied in the treatment praxis. Often, it is very hard for practical reasons to reproduce the simultaneous full scope and cellular constituents of OA disease state in vitro. Hence, most in vitro OA models are designed to focus on the late disease stages with a substantial limitation in the evaluation of disease mechanisms during early stages that may be crucial in targeting that disease.

## Abbreviations

ACAN: Aggrecan
CD14: Cluster of differentiation 14
cDNA: Complementary DNA
COL1A1: Collagen 1
COL2A1: Collagen 2
DMARDs: Disease-modifying anti-rheumatic drugs
HA: Hyaluronic acid
IL-1β: Interleukin-1 beta
IL-6: Interleukin-6
iNOS: Inducible nitric oxide synthase
LPS: Lipopolysaccharide
MDM: Monocyte-derived-macrophages
MMP: Matrix Metalloproteinases
NSAIDs: Non-steroidal anti-inflammatory drugs
OA: Osteoarthritis
PBS: Phosphate buffered saline
PCR: Polymerase chain reaction
rM0: Resting M0 macrophages
TNF-α: Tumor necrosis factor alpha
